# A metabolic comparison of GIPR agonism versus GIPR antagonism in male mice

**DOI:** 10.1111/dom.70300

**Published:** 2025-11-24

**Authors:** Iona Davies, Alexandra Turland, Hanh Duyen Tran, Carissa Wong, Olivier Cahn, Cecilia Dunsterville, Yichang Sun, Yilin Xiao, Kevin G. Murphy, Stephen R. Bloom, Ben Jones, Tricia M. M. Tan

**Affiliations:** ^1^ Section of Endocrinology and Investigative Medicine Imperial College London London UK

**Keywords:** animal pharmacology, antiobesity drug, drug mechanism, GIP

## Abstract

**Aims:**

Targeting the glucose dependent insulinotropic polypeptide receptor (GIPR) is of growing interest for treating type 2 diabetes and obesity, though the optimal approach remains unclear. Both GIPR agonism and antagonism, respectively, incorporated into drugs like tirzepatide and maridebart cafraglutide, have paradoxically both shown significant weight loss effects in humans.

**Materials and methods:**

In this study, the metabolic impacts of a GIPR agonist (GIP108) and antagonist (NN‐GIPR‐Ant) were evaluated in lean and high‐fat diet (HFD)–induced obese male mice. We assessed the impacts on food intake, body weight, glucose and insulin tolerance, liver triglyceride levels, bone markers and adipose tissue lipolytic gene expression.

**Results:**

In lean mice, neither peptide affected food intake or body weight, but GIP108 improved glucose tolerance. In obese mice, both agents reduced food intake and body weight, with NN‐GIPR‐Ant producing more sustained appetite suppression. Energy expenditure remained unchanged, as weight loss matched that of pair‐fed controls. GIP108 improved glucose tolerance independently of weight loss, whereas NN‐GIPR‐Ant reduced insulin sensitivity compared to pair‐fed controls. Both treatments slightly increased liver triglyceride content compared to their pair‐fed controls, and no treatment significantly affected plasma bone marker levels. Finally, NN‐GIPR‐Ant reduced the expression of adipose tissue lipolytic genes.

**Conclusions:**

Our data highlights the distinct metabolic effects of GIPR agonism and antagonism, offering insights for their future application in personalised metabolic disease treatments. Further human studies are needed to understand the long‐term metabolic impacts of these therapies.

## 1 INTRODUCTION

In recent years, there has been a resurgence of interest in targeting the glucose‐dependent insulinotropic polypeptide receptor (GIPR), alongside the glucagon‐like peptide 1 receptor (GLP‐1R), for the treatment of metabolic disease. Interestingly, there is evidence that both activating and inhibiting GIPR can be metabolically beneficial. Both GIPR knockout mice and GIP over‐expressing mice are protected from high‐fat diet–induced obesity,^1^, ^2^, ^3^, ^4^, ^5^ and both GIPR agonists and GIPR antagonists induce moderate weight loss in preclinical obesity models.^6^, ^7^, ^8^ This paradox extends to man, with long‐acting GIPR agonist LY3537021 driving moderate weight loss in humans,^9^ despite loss‐of‐function GIPR variants being associated with increased BMI.^10^ Importantly, both strategies have been translated into efficacious obesity pharmacotherapies when combined with GLP‐1R agonism; tirzepatide, a marketed GIPR/GLP‐1R dual agonist and maridebart cafraglutide, a GIPR antagonist/GLP‐1R agonist currently in phase III clinical trials.^11^, ^12^

While previous studies have described metabolic consequences of either GIPR agonist or antagonist treatment in isolation,^2^, ^6^, ^7^, ^13^, ^14^ an in‐depth side‐by‐side comparison of each approach of GIPR targeting has yet to be conducted. Thus, the focus of our study was to comprehensively analyse the metabolic benefits and drawbacks of GIPR agonist versus GIPR antagonist treatment in a lean mouse model, and a high‐fat‐diet (HFD)–induced obese mouse model with impaired glucose tolerance.

## 2 RESULTS

### 2.1 Selecting an appropriate GIPR agonist and GIPR antagonist

Our first aim was to select appropriate agonist and antagonist GIPR ligands for in vivo study.

**FIGURE 1:**
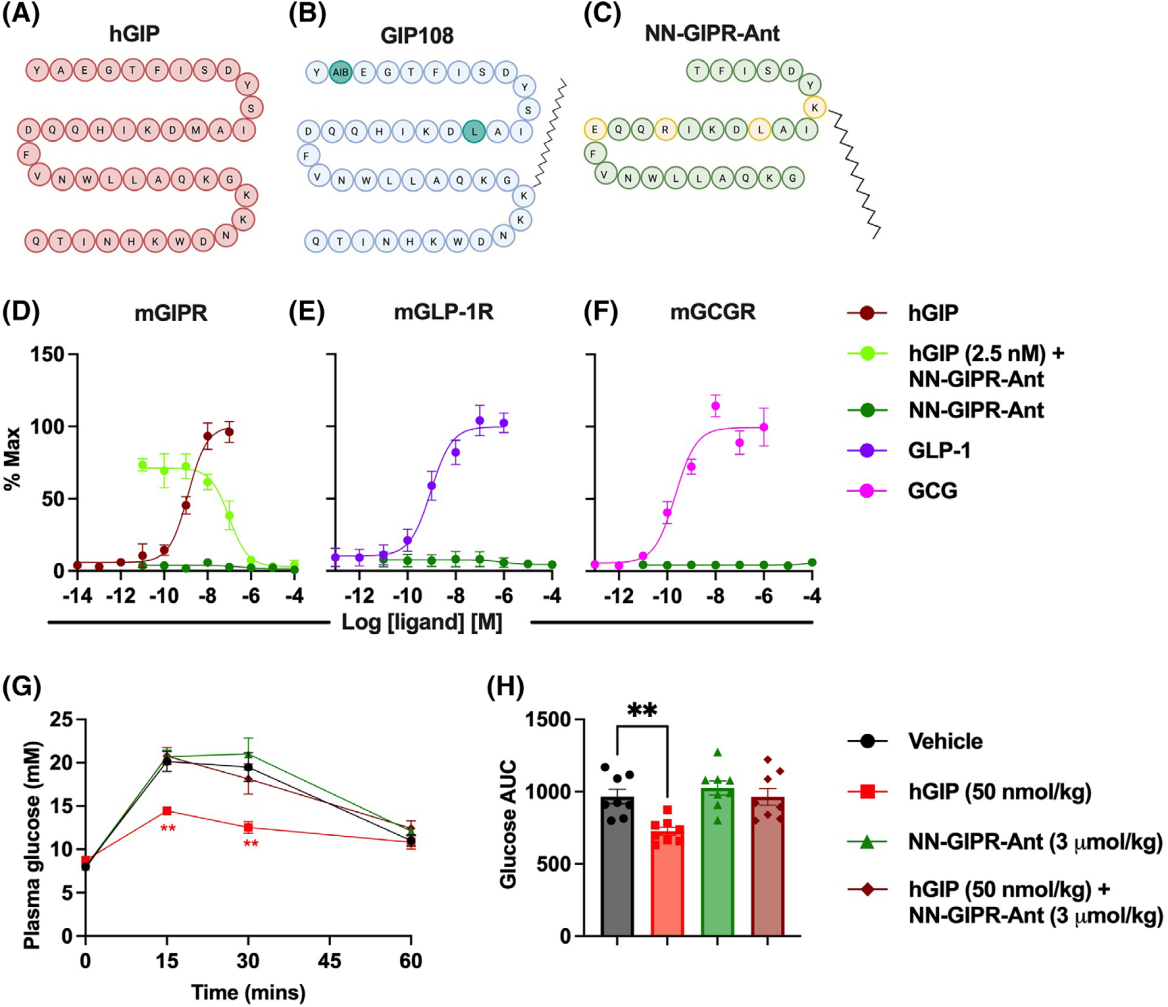
NN‐GIPR‐Ant is a specific mouse GIPR antagonist. (A–C) Amino acid sequences of hGIP (A), GIP108 (B) and NN‐GIPR‐Ant (C). AIB = 2‐aminoisobutyric acid. Side chains denote fatty acid tails. (D–F) cAMP measurements in AD293 cells transiently transfected with the mGIPR (D), mGLP‐1R (E) and mGCGR (F) and stimulated for 30 min with the indicated peptide (*n* = 3), with three parameter curve fits plotted. (G, H) 60‐min intraperitoneal glucose tolerance test in lean mice (*n* = 8). (G) Plasma glucose over time with glucose ± hGIP injected at *t* = 0 and NN‐GIPR‐Ant injected at *t* = −120 min, analysed using two‐way ANOVA with time and subgroup as co‐variables. (H) Glucose AUC derived from the time course in (H), analysed using a one‐way ANOVA. Dunnett's test was used to correct for multiple comparisons against vehicle control. All values are displayed as mean ± SEM. **p < 0.01. The colour of the star denotes which group is statistically different compared to vehicle control.

GIP108, our previously reported GIPR agonist, was selected as it is a potent, selective and long‐acting agonist of the mouse GIPR.^15^ For the antagonist we used a long‐acting GIPR antagonist, named throughout as NN‐GIPR‐Ant, previously characterised by Yang et al.^7^ A comparison of the amino acid structures of these peptides compared to hGIP(1‐42) is shown in Figure 1A–C. We first confirmed the actions of NN‐GIPR‐Ant as a mouse GIPR antagonist, performing in vitro cAMP assays in AD293 cells, a human embryonic kidney cell line commonly used in G‐protein coupled receptor research due to rapid division and ease of receptor transfection. Increasing doses of NN‐GIPR‐Ant were able to block hGIP's ability to stimulate cAMP in AD293 cells expressing the mouse GIPR (Figure 1D), with 1 μM of NN‐GIPR‐Ant fully blocking the effects of 2.5 nM of hGIP. Moreover, NN‐GIPR‐Ant did not display any partial GIPR agonist activity (Figure 1D), nor did it stimulate cAMP production at the mouse GLP‐1R (Figure 1E) and mouse GCGR (Figure 1F). NN‐GIPR‐Ant was confirmed as a GIPR antagonist in vivo, fully blocking the ability of hGIP (50 nmol/kg) to improve glucose tolerance during an intraperitoneal glucose tolerance test in lean mice (Figure 1G,H).

### 2.2 GIP108 and NN‐GIPR‐Ant elicit distinct glucoregulatory responses in lean mice

**FIGURE 2:**
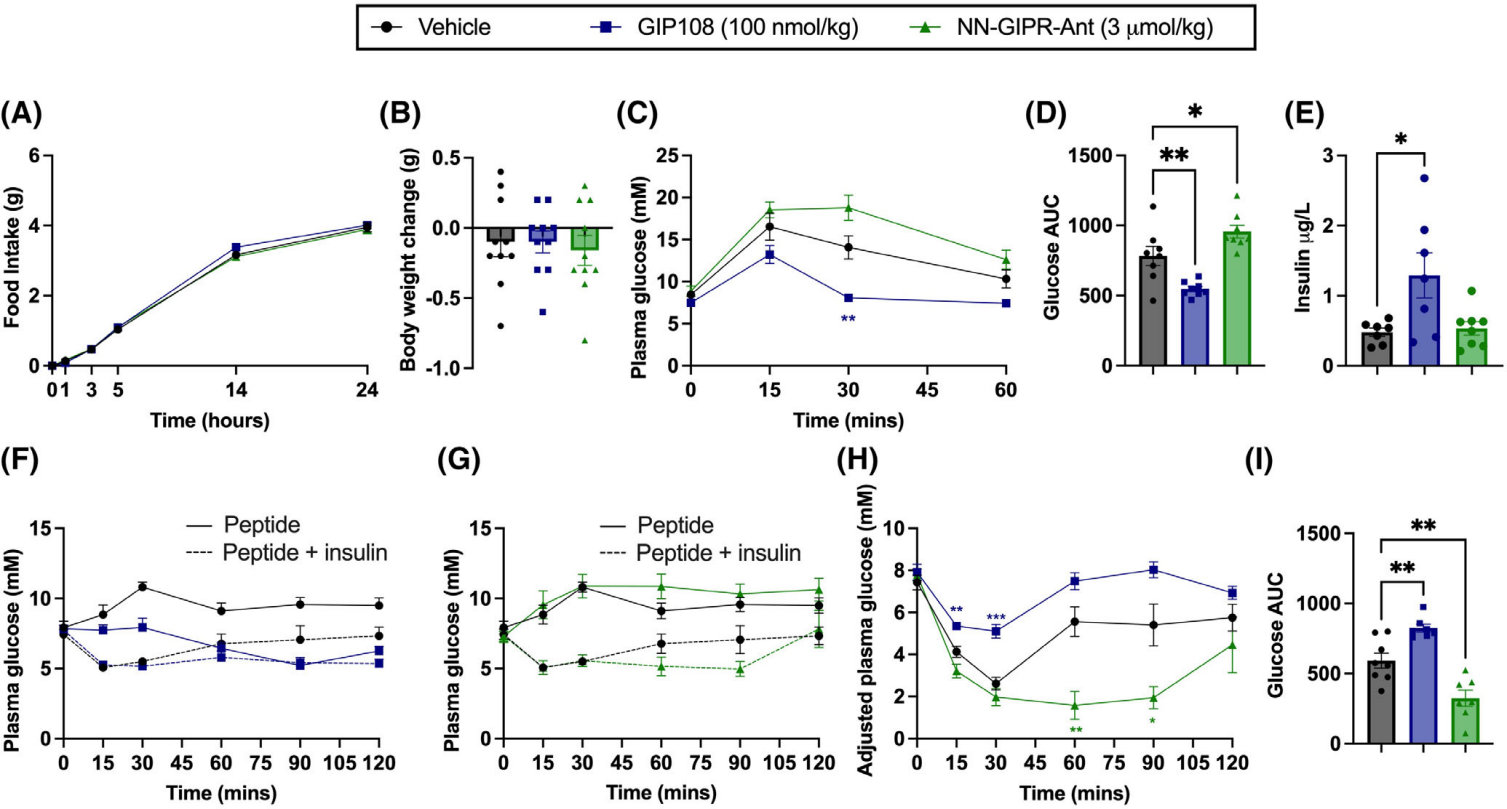
GIP108 and NN‐GIPR‐Ant elicit distinct glucoregulatory responses in lean mice. Throughout this panel, vehicle, GIP108 (100 nmol/kg) and NN‐GIPR‐Ant (3 μmol/kg) are administered acutely to lean mice. (A, B) Cumulative food intake (g) (A) and body weight change (g) (B) in lean mice (*n* = 10) following subcutaneous peptide injection. (C, D) 60‐min intraperitoneal glucose tolerance test in lean mice (*n* = 8), with glucose injected at *t* = 0 and peptides injected subcutaneously at *t* = −120 min. (C) Plasma glucose time course and (D) glucose AUC derived from time course in (C). (E) Plasma insulin (μg/L) levels in lean mice (*n* = 7–8) at *t* = 15, with glucose administered at *t* = 0 and peptides injected subcutaneously at *t* = −120 min. (F–I) 120‐min intraperitoneal insulin tolerance test in lean mice (*n* = 7–8), with peptides and insulin injected at *t* = 0. (F, G) Plasma glucose time course with solid lines representing peptide administered without insulin, and dotted lines representing peptide administered with insulin. (F) Vehicle vs. GIP108 (100 nmol/kg) and (G) Vehicle vs. NN‐GIPR‐Ant (3 μmol/kg). (F) and (G) share the same vehicle group. (H) Plasma glucose time course for peptide + insulin groups, adjusted for glucose changes when peptide is administered without insulin. (I) Glucose AUC derived from time course in (H). Changes in food intake and blood glucose have been analysed using a two‐way ANOVA with time and subgroup as covariables. Changes in body weight, plasma insulin and glucose AUC have been analysed using a one‐way ANOVA. Dunnett's test was used to correct for multiple comparisons against vehicle control. All values are displayed as mean ± SEM. *p < 0.05, **p < 0.01, ***p < 0.001. The colour of the star denotes which group is statistically different compared to vehicle control.

Following selection of appropriate peptides, we evaluated their metabolic impact following acute subcutaneous administration to lean chow‐fed male mice. Peptide doses (100 nmol/kg for GIP108, 3 μmol/kg for NN‐GIPR‐Ant) were selected following pilot data showing matched anorectic capacity in HFD‐induced obese mice (Figure S1, Supporting Information). For further details on dose selection, see “Supplementary Note” section therein. In this lean model, neither GIP108 nor NN‐GIPR‐Ant reduced appetite (Figure 2A) or body weight (Figure 2B). During an IPGTT, glucose tolerance was significantly improved by GIP108 administration (Figure 2C,D), mirrored by significantly increased glucose‐stimulated plasma insulin levels compared to vehicle control in a separate study (Figure 2E). Glucose tolerance was moderately worsened by NN‐GIPR‐Ant (Figure 2C,D), an unexpected finding given that GIPR KO mice do not display impairments in glucose tolerance following intraperitoneal glucose injection.^16^ However, glucose‐stimulated plasma insulin levels following NN‐GIPR‐Ant administration were not significantly different from vehicle control (Figure 2E). These effects were then studied at a cellular level via measurement of cAMP production in dispersed pancreatic islets from lean mice using the cAMP biosensor cADDis; GIP108 significantly increased cAMP production whereas NN‐GIPR‐Ant reduced baseline cAMP levels, suggesting NN‐GIPR‐Ant may have some action as an inverse agonist at the pancreatic GIPR, which is thought to display a significant degree of constitutive activity (Figure S2).^17^

We next investigated the effects of GIP108 and NN‐GIPR‐Ant administration on insulin sensitivity. To adjust for the insulinotropic effects of GIP108, mice were given either peptide alone or peptide in combination with insulin (Figure 2F,G). This enabled plasma glucose values to be adjusted for the effects on insulin production of the peptide alone (Figure 2H,I). Interestingly, adjusted plasma glucose was raised following GIP108 administration and lowered following NN‐GIPR‐Ant administration compared to vehicle. However, we predict that these observations are not through effects on insulin sensitivity but instead reflect GIP's ability to potently stimulate glucagon secretion in hypoglycaemia,^18^ a mechanism activated by GIP108 and blocked by NN‐GIPR‐Ant.

### 2.3 GIP108 and NN‐GIPR‐Ant both induce weight loss in HFD‐induced obese mice

**FIGURE 3:**
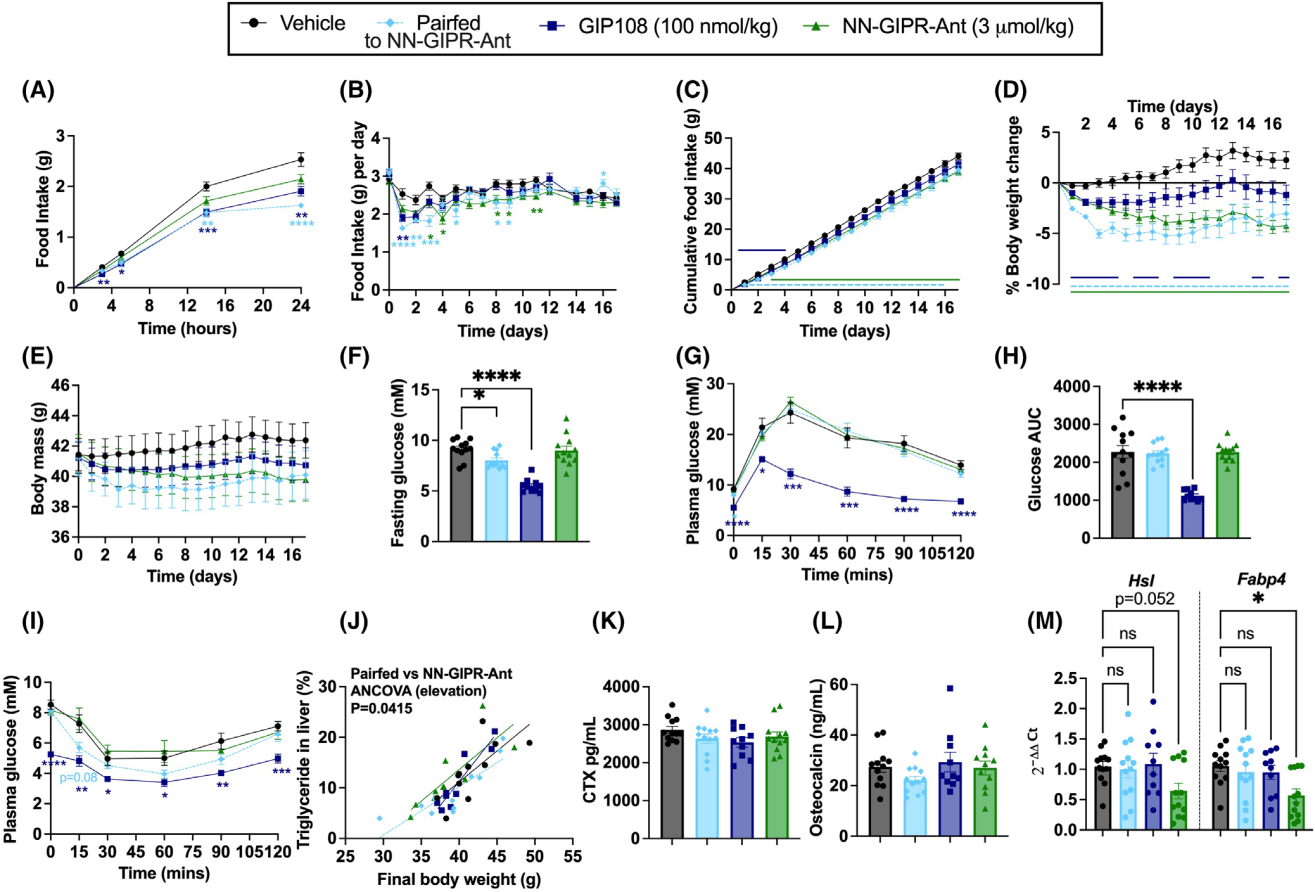
GIP108 and NN‐GIPR‐Ant both reduce body weight in HFD induced obese mice. HFD‐induced obese mice received daily afternoon subcutaneous injections of saline (*n* = 12), GIP108 (100 nmol/kg) (*n* = 10) and NN‐GIPR‐Ant (3 μmol/kg) (*n* = 12) for 17 days. An additional 12 mice were pair‐fed to NN‐GIPR‐Ant. (A) Acute food intake (g) over 24 h after first injection, (B) food intake (g) per day over 17 days, (C) cumulative food intake (g) over 17 days, (D) % change in body weight over 17 days, (E) body mass (g) over 17 days, (F) fasting glucose (mM) on Day 13/14, 2 h after daily peptide injection, (G) plasma glucose (mM) and (H) glucose AUC measured during intraperitoneal glucose tolerance test on Day 13/14, 2 h after daily peptide injection with glucose administered at *t* = 0, (I) plasma glucose measured during an intraperitoneal insulin tolerance test on Day 15/16, 2 h after daily peptide injection with insulin administered at *t* = 0, (J) relationship between final body weight and triglyceride in liver (%) following 17 days of daily injection (1 outlier excluded per group), (K) plasma CTX (pg/mL) and (L) plasma osteocalcin (ng/mL) (1 outlier excluded for NN‐GIPR‐Ant), both measured following 17 days of daily injection, (M) inguinal adipose tissue mRNA expression of hormone sensitive lipase (*Hsl*) and fatty acid binding protein 4 (*Fabp4*), expressed relative to housekeeping gene, measured following 17 days of daily injection. Food intake, body weight and plasma glucose measurements over time have been analysed using a two‐way ANOVA with time and subgroup as covariables, with Dunnett's test applied to correct for multiple comparisons against vehicle control. Gene expression has been analysed using a two‐way ANOVA with gene and subgroup as covariables, with Dunnett's test applied to correct for multiple comparisons against vehicle control. Fasting glucose, glucose AUC and plasma CTX/osteocalcin levels have been analysed using a one‐way ANOVA with Dunnett's test applied to correct for multiple comparisons against vehicle control. % triglyceride in liver has been analysed using ANCOVA between the pair‐fed group and NN‐GIPR‐Ant group, with final body weight as a covariable. All values are displayed as mean ± SEM. Lines and stars indicate statistically significant differences. *p < 0.05, **p < 0.01, ***p < 0.001, ****p < 0.0001. The colour of the star or line denotes which group is statistically different compared to vehicle control.

We next studied the effects of GIPR agonism versus GIPR antagonism in a HFD‐induced obese mouse model with impaired glucose tolerance. GIP108 and NN‐GIPR‐Ant were administered daily via subcutaneous injection for 17 days. An additional 12 mice were pair‐fed to NN‐GIPR‐Ant. As expected from previous studies of GIPR agonists and GIPR antagonists in HFD‐induced obese mice, both GIP108 and NN‐GIPR‐Ant significantly reduced food intake and body weight throughout the 17 days of daily administration (Figure 3A–E). Interestingly, we observed temporal differences in food intake reduction between both methods of receptor targeting. While GIP108 was more anorectic than NN‐GIPR‐Ant after first administration (Figure 3A), this anorexia waned over time, with the GIP108 group consuming the same as the vehicle group by Day 6 (Figure 3B,C). In contrast, NN‐GIPR‐Ant sustained moderate food intake reduction throughout the entirety of the study (Figure 3B,C). This is reflected in the greater weight loss observed following NN‐GIPR‐Ant than GIP108 (Figure 3D). We also observed matched weight loss between NN‐GIPR‐Ant and its pair‐fed group (Figure 3D), indicating NN‐GIPR‐Ant had not significantly impacted energy expenditure.

As seen previously,^15^ chronic GIP108 administration significantly improved fasting glucose (Figure 3F) and glucose tolerance (Figure 3G,H). NN‐GIPR‐Ant did not reduce fasting glucose nor improve glucose tolerance. However, in contrast to our studies in lean mice, NN‐GIPR‐Ant did not worsen either of these parameters. An IP insulin tolerance test was also performed: while the NN‐GIPR‐Ant pair‐fed group displayed modest improvements in insulin sensitivity compared to vehicle control (*p* = 0.08 at *t* = 15), this was not seen for NN‐GIPR‐Ant (Figure 3I).

Interestingly, while neither GIP108 nor NN‐GIPR‐Ant administration significantly impacted percentage liver triglyceride content compared to vehicle (Figure S3), NN‐GIPR‐Ant treated animals had greater percentage liver triglyceride content than their pair‐fed control, assessed via ANCOVA with body weight as a covariate (Figure 3J). Oil Red O staining intensity reflected percentage liver triglyceride content in a subset of stained livers (Figure S4). In addition, as GIP increases osteoblastic and suppresses osteoclastic activity,^19^ we tested for biomarkers of bone turnover in terminal plasma samples. GIP108 administration did not significantly affect plasma C‐terminal telopeptide (CTX) levels (Figure 3K) or plasma osteocalcin levels (Figure 3L) following 17‐day administration. We also investigated the mRNA expression of key lipolytic markers hormone sensitive lipase (*Hsl*) and fatty acid binding protein 4 (*Fabp4*) in inguinal adipose tissue following 17‐day peptide administration. Chronic NN‐GIPR‐Ant administration resulted in a significant decrease in expression of *Fabp4* and a trend towards a decrease in expression of *Hsl* (*p* = 0.052), both compared to vehicle control, suggesting GIPR antagonism had decreased adipose tissue lipolysis (Figure 3M).

Finally, we performed an additional post hoc analysis, selecting a subset of the restrictedly fed group (*n* = 7) that was matched for food intake to the GIP108 group (Figure S5). The following observations were made: (1) improvements in glucose tolerance following GIP108 administration were independent of weight loss as they were not observed in the pair‐fed group (Figure S5G,H), (2) as with NN‐GIPR‐Ant, the pair‐fed group displayed improvements in insulin sensitivity that were not observed in the GIP108 group (Figure S5I) and (3) GIP108 trended towards a modest increase in percentage liver triglyceride content compared to its pair‐fed control, assessed via ANCOVA with body weight as a covariate (*p* = 0.09) (Figure S5J).

## 3 DISCUSSION

We are in an era of mass development of efficacious obesity pharmacotherapies acting on multiple incretin and allied receptors. Differentiating the metabolic effects of targeting each incretin receptor is crucial to the personalisation of obesity treatment. To this end, this study was focused on understanding the metabolic benefits and drawbacks of both GIPR agonism and antagonism. Both are valid pharmacological strategies integrated into the class‐leading GIPR/GLP‐1R dual agonist peptide tirzepatide, and the investigational GIPR antagonist/GLP‐1R agonist maridebart cafraglutide.^11^, ^12^ Here, we have confirmed the modest anorectic effects of a long‐acting GIPR agonist and GIPR antagonist in HFD‐induced obese mice. Moreover, we studied several other metabolic pathways affected by GIPR targeting including glucose tolerance, insulin sensitivity, liver triglyceride content and markers of bone health.

A number of observations were made with regard to energy balance. First, unlike GLP‐1 and other anorectic hormones,^20^ GIP108 and NN‐GIPR‐Ant did not impact body weight or food intake in lean mice. This observation has been made for another GIPR antagonist previously, with authors hypothesizing that GIPR antagonists act through counteracting HFD‐induced leptin resistance.^21^ An alternative explanation could be that GIPR targeting produces a relatively weak anorectic signal and thus appetite reduction is only possible in models where appetite regulation is disrupted, for example, HFD‐induced obesity. Regardless of the mechanism, this finding has important implications for the interpretation of studies administering GIPR agonists/antagonists (or dual receptor targeting ligands with GIPR activity) to *Gipr*/*Glp1r* knockout mice, as these models are resistant to HFD‐induced weight gain and may therefore have inherent resistance to GIPR action irrespective of their receptor status. In other words, if GIPR targeting does not impact appetite in a receptor knockout model, this does not necessarily mean that the peptide in question acts to reduce appetite through that knocked out receptor but could be due to baseline differences in body weight‐related GIPR sensitivity.

Second, while GIP108 and NN‐GIPR‐Ant both reduced appetite and body weight in HFD‐induced mice, GIP108 was most effective acutely whereas NN‐GIPR‐Ant's anorectic effect was sustained throughout the 17 days of daily administration. While we did not perform a direct pharmacokinetic comparison between agents, it is unlikely this is the cause as peptides were administered daily throughout the study. Instead, this could reflect agonist‐induced GIPR desensitisation following chronic exposure to GIP108, extensively explored previously by our group at the pancreas.^15^ As NN‐GIPR‐Ant is a GIPR antagonist, receptor desensitisation following chronic NN‐GIPR‐Ant exposure would be unexpected. With that being said, how phenotypic differences arise from agonist‐induced GIPR desensitisation (waning food intake reduction) versus true receptor antagonism (prolonged food intake reduction), both phenomena that prevent physiological GIPR signalling, remains unknown. A comparison of the anorectic effects of chronic administration of a GIPR antagonist versus a GIPR agonist with a reduced desensitisation tendency, for example, a G‐protein biased GIPR agonist described in Rodriguez et al.,^22^ would be an interesting area of future study.

Third, we detected no impact of GIP108 or NN‐GIPR‐Ant administration on energy expenditure, as weight loss was equivalent to pair‐fed controls, a finding consistent with another study investigating GIPR agonism.^2^ This is surprising given the robust increase in adipose tissue lipolysis observed by Regmi et al. following acute GIPR agonism,^23^ and the decreased mRNA expression of lipolytic genes in inguinal adipose tissue following chronic GIPR antagonism observed in this study. The role of GIPR agonism versus antagonism on energy expenditure is worthy of further investigation, perhaps as a dedicated study assessing energy expenditure through use of metabolic cages.

The most striking difference between GIP108 and NN‐GIPR‐Ant administration was the effect on glucose tolerance. In both lean and HFD‐induced obese mice, GIP108 produced marked improvements in glucose tolerance whereas NN‐GIPR‐Ant had no impact. It is currently unresolved as to how GIPR agonists and GIPR antagonists can have the same effect on appetite, but different effects on glucose tolerance. Killion et al. proposed a theory of differential rates of receptor desensitisation, whereby central GIPRs desensitise in response to agonist stimulation more readily than pancreatic GIPRs, and thus a GIPR agonist acts as an antagonist in the CNS but not in the pancreas.^13^ While not decisively disproven, evidence against this theory comes from observations of the acute anorectic effects of GIPR agonists, as well as the extensive agonist‐induced GIPR desensitisation in pancreatic beta cells.^15^ An alternative explanation was recently proposed by Gutgesell et al., who postulated that GIPR agonists and GIPR antagonists engage different central pathways to reduce appetite, with GIPR antagonists reliant on functional GLP‐1R signalling to reduce appetite.^24^ Concerning this, it is unclear why a GIPR antagonist has a CNS phenotype despite there being a lack of evidence for GIP production in the brain.^4^ In this study we showed that GIPR antagonism reduces basal levels of cAMP in dispersed pancreatic islet cells, thus providing evidence that it can reduce the constitutive activity of the receptor. It is therefore conceivable that a GIPR antagonist might suppress such constitutive activity in neuronal populations, but to date this has not been directly demonstrated.

Another important focus of this study was the effect of GIP108 and NN‐GIPR‐Ant administration on insulin sensitivity. Regmi et al. have previously demonstrated the acute insulin sensitising effect of GIP in human adipocytes, increasing adipocyte glucose uptake both ex vivo and in vivo following acute administration,^23^ and Samms et al. showed that chronic GIPR agonism resulted in an increased glucose infusion rate during a euglycaemic–hyperinsulinaemic clamp in mice.^25^ We assessed insulin sensitivity via IPITT, a cruder but higher throughput method, and were unable to reveal any insulin sensitising effects of GIP108. This is potentially due to GIPR agonist induced glucagon production being more pronounced in hypoglycaemia than euglycaemia.^26^ While the relative glucose lowering effects of NN‐GIPR‐Ant in an acute setting in lean mice likely reflected blocked GIP‐induced glucagon production, the effects of NN‐GIPR‐Ant on insulin sensitivity following chronic administration provided greater insight. While the pair‐fed group displayed lowered plasma glucose in the early stages of the IPITT, this was not observed in mice administered NN‐GIPR‐Ant. This suggests that the chronic administration of NN‐GIPR‐Ant had neutralised any improvements in insulin sensitivity that would be provided by weight loss, a finding in line with the earlier studies of Samms et al. and Regmi et al.^23^, ^25^

We also investigated the effects of GIPR agonism and antagonism on liver triglyceride content. This was explored following evidence from a human study of increased liver fat after a 6‐day GIP infusion.^27^ Interestingly, in our study there were subtle increases in liver fat compared to the pair‐fed group following NN‐GIPR‐Ant administration, and a trend for the same observation following GIP108 administration. There is no obvious explanation for this finding: a dedicated study investigating the effects of non‐anorectic doses of GIP108/NN‐GIPR‐Ant on liver triglyceride levels at multiple time points could provide further insight. Finally, we showed that chronic GIP108 administration did not lower plasma CTX levels nor raise plasma osteocalcin levels. Given evidence that GIP administration acutely lowers CTX levels in both rodent models and humans,^28^, ^29^ perhaps this is a reflection of chronic administration and it would be interesting to measure these markers at earlier time points.

While preclinical in nature, this study provides clues as to how GIPR agonism and antagonism might be positioned for personalisation of obesity treatment. If a non‐diabetic patient displays suboptimal weight loss in response to tirzepatide treatment, switching to a GLP‐1R agonist/GIPR antagonist approach may enhance weight loss. While GIPR antagonists require higher dosing to achieve an anorectic effect, this has not prevented GIPR antagonism from being pursued as a therapeutic strategy, evidenced by Amgen's high‐affinity GIPR antagonist antibody with GLP‐1R agonist conjugate arms maridebart cafraglutide currently in phase III trials,^12^ and Antag therapeutics have initiated phase I trials of their peptide GIPR antagonist AT‐7687.^8^ Agents incorporating GIPR agonism might optimally benefit patients with type 2 diabetes through improvements in both glucose tolerance and insulin sensitivity. Finally, perhaps someone with fatty liver disease would benefit more from a GLP‐1R/GCGR co‐agonist than an agent targeting GIPR, if equally efficacious for weight loss. A clear limitation of this study is that it is unlikely that a GIPR agonist or antagonist will be given alone for the treatment of obesity/diabetes as weight loss is modest compared to GLP‐1R agonism. While this initial study is the simplest way of deciphering the individual metabolic benefits of GIPR agonism versus GIPR antagonist, a thorough metabolic comparison of a combinatorial approach (including assessments beyond just body weight) is vital for the advancement of personalised obesity research.

In conclusion, this study characterises the metabolic effects of GIPR agonist and antagonist administration in both lean and HFD‐induced mice, providing novel insights highly relevant to the personalisation of obesity treatments.

## FUNDING INFORMATION

I.D. was funded via a Medical Research Council Doctoral Training Partnership (MR/N014103/1). K.G.M. is supported by Diabetes UK (18/0005886, 20/0006295), the BBSRC (BB/W001497/1, BB/X017273/1), the MRC (MR/Y013980/1) and the Wellcome Trust (310835/Z/24/Z). B.J. acknowledges funding support from the Medical Research Council (MR/Y00132X/1 and MR/X021467/1), the Wellcome Trust (301619/Z/23/Z), Diabetes UK, the Eli Lilly LRAP programme, and Metsera Inc. T.M.M.T. is supported by the NIHR BRC and grants from MRC, Diabetes UK and NIHR.

## CONFLICT OF INTEREST STATEMENT

I.D., C.W., O.C., Y.S., Y.X., C.D. and K.G.M. have nothing to declare. A.T. and H.D.T. have received funding support from Metsera Inc. S.R.B. is employed by Zihipp Ltd. S.R.B. is an inventor on patent #PCT/GB2024/050504 licensed to IP2IPO INNOVATIONS LIMITED. B.J. consults for Metsera Inc. B.J. has received grant support from Eli Lilly and Company, Sun Pharmaceutical Industries Ltd., and Metsera Inc. T.M.M.T. has previously consulted for Zihipp Ltd.

## Supporting information


**Data S1.** Supporting Information.

## Data Availability

The data that support the findings of this study are available from the corresponding author upon reasonable request.
