# Supplementary material for: A metabolic comparison of GIPR agonism versus GIPR antagonism in male mice
Source: Diabetes Obes Metab. 2025 Nov 24;28(2):1160–7. doi: 10.1111/dom.70300 (PMC12803606; doi:10.1111/dom.70300)
Supplement: Supplementary file 1 — Data S1. Supporting Information. [file DOM-28-1160-s001.docx]

**Supplementary Appendix**

**Materials and methods:**

**Peptides**

GLP-1(7–36)·NH_2_, glucagon(1–29) and human GIP(1–42) were purchased from Bachem, Switzerland; GIP108 and NN-GIPR-Ant from WuXi AppTec, China. GIP108 is a modified version of human GIP, with amino acid substitutions at position 2 (alanine to 2-aminoisobutyric acid) and position 14 (methionine to leucine), with a 20-carbon dicarboxylic acid added at position 32^1^. NN-GIPR-Ant was synthesised in accordance with the sequence published by Yang et al ^2^ – compared to the human GIP sequence, the first 4 and last 11 amino acids are removed and amino acid modifications are found at position 11 (serine to lysine), position 14 (methionine to leucine), position 18 (histidine to arginine, as found in the mouse GIP sequence) and position 21 (aspartic acid to glutamic acid), with a 16-carbon carboxylic acid added at position 11. All Wuxi compounds were synthesized using solid phase peptide synthesis and purified using reverse phase high performance liquid chromatography, resulting in >85% peptide purity.

**Cell culture**

AD-293 cells (Agilent, CA, USA) were maintained in Dulbecco’s modified medium (DMEM, Thermo Fisher Scientific, UK) with 1% penicillin/streptomycin (Sigma, Welwyn Garden City, UK) and 10% foetal bovine serum (Thermo Fisher Scientific, UK).

**Plasmids**

Plasmids encoding full length, wild-type mouse GIPR, GLP-1R or glucagon receptor (GCGR) in the pcDNA5/FRT vector (Thermo Fisher Scientific, UK) were custom synthesized by Genewiz, UK.

**HTRF cAMP assays**

These were as previously described^1^. Briefly, AD-293 cells were transfected with desired plasmid DNA (1 µg of DNA + Lipofectamine 2000, Thermo Fisher Scientific, UK) 24 hours prior to cAMP accumulation assays performed using the cAMP-Gs Dynamic 2 kit (Cisbio, Codolet, France), as per manufacturer’s protocol.

**Animals**

All animal procedures were approved by the British Home Office under the UK Animals (Scientific Procedures) Act 1986, and Amendment Regulations, 2012 and approved by the Imperial College London Animal Welfare and Ethical Review Body. Wild type male C57BL/6J mice (8-14 weeks; Charles River, UK) were used. Mice were housed in individually ventilated cages with a standard 12:12 hour light-dark cycle. Unless fasted, mice had free access to water and food pellets (lean mice: RM1 – Special Diet Services, UK; high fat diet (HFD) induced obese mice: 60% kcal fat diet, D12492, Research Diets, Inc., NJ USA). For food intake studies, mice were singly housed. For all other studies, mice were group housed (5 mice per cage). For studies involving HFD-induced obese mice, mice were maintained on HFD for 6-10 weeks prior to initiation of studies. For the main chronic study involving HFD-induced obese mice, 7 weeks was identified as an adequate time to induce obesity (Figure 3E) and impaired glucose tolerance (Figure 3G). Mice that were less than 35 g were excluded from the main study prior to initiation.

**Peptide dosing**

In all *in vivo* studies, mice were randomised into groups by body weight. Mice received a 100 µL subcutaneous injection of saline (0.9% sodium chloride) or peptide at a specified dose, diluted in saline. Peptide dosing was in nmol/kg of total body weight.

**Acute studies in lean mice**

All studies conducted in lean mice were following single peptide administration.

To confirm NN-GIPR-Ant acted as a GIPR antagonist *in vivo*, an intraperitoneal glucose tolerance test was conducted, with NN-GIPR-Ant administered subcutaneously at 12:00 and hGIP administered with glucose at 14:00.

For metabolic comparison of GIP108 vs NN-GIPR-Ant in lean mice, a food intake study, insulin bleeds, intraperitoneal glucose tolerance test and intraperitoneal insulin tolerance test were conducted. For acute feeding studies, ad libitum fed mice were injected at 17:00 and food intake monitored at 1, 3, 5, 14 and 24 hours. Body weight was assessed at 24 hours.

**Acute and chronic studies in HFD-induced obese mice:**

Multiple pilot studies in HFD-induced obese mice were conducted to identify comparable anorectic doses of GIP108 and NN-GIPR-Ant. Across all studies, mice received their first injection at 17:00 with food intake monitored at 1, 3, 5, 14 and 24 hours. For all chronic studies, mice were then injected daily between 16:00 and 17:00, with food intake and body weight also measured daily.

In the first pilot study, HFD-induced obese mice received a daily subcutaneous injection of saline, GIP108 (10 nmol/kg), GIP108 (30 nmol/kg) or GIP108 (100 nmol/kg) (n=5-8/group) for 18 days. In the second pilot study, HFD-induced obese mice received a daily subcutaneous injection of saline, GIP108 (100 nmol/kg) or NN-GIPR-Ant (1.5-3 µmol/kg) (n=7-8/group) for 20 days. For the first 14 days of the study, NN-GIPR-Ant was administered at 1.5 µmol/kg. For the remaining 6 days of the study, NN-GIPR-Ant was administered at 3 µmol/kg. In the third pilot study, HFD-induced obese mice received an acute injection of saline, GIP108 (100 nmol/kg) or NN-GIPR-Ant (3 µmol/kg) (n=4-5/group).

Following identification of suitable peptide doses, HFD-induced obese mice (mean starting weight = 41.3 g) received a daily subcutaneous injection of saline, GIP108 (100 nmol/kg) or NN-GIPR-Ant (3 µmol/kg) (n=10-12/group) for 17 days. Another group of mice with restricted access to food (n=12) were injected daily with saline. These mice were initially pair-fed to the average food intake of the GIP108 group. However, a proportion of these mice did not eat all food given. Thus, for the remainder of the study, mice were individually restrictively fed to account for variation in food intake. By the end of the 17-day study, the restrictively fed mice had consumed the same amount as the NN-GIPR-Ant group. In an additional post hoc analysis, 7 restrictively fed mice had eaten the same amount as the GIP108 group.

An intraperitoneal glucose tolerance test was conducted on day 13 for mice 1-24, and day 14 for mice 25-48. An intraperitoneal insulin tolerance test was conducted on day 15 for mice 1-24 and day 16 for mice 25-48. On these days, mice received their daily injection of peptide at midday. Mice were culled on day 17 via decapitation to allow for collection of blood for plasma analysis. Liver lobes and subcutaneous adipose tissue were dissected and snap frozen in liquid nitrogen.

**Intraperitoneal glucose tolerance test**

Mice were fasted at 08:00. Long-acting peptides were administered via subcutaneous injection at 12:00 and glucose administered at 14:00. 20% glucose was administered with the injection volume adjusted to the body weight of the mouse such that each mouse received glucose at 2 g/kg. Blood glucose measurements were taken via tail venesection at 0, 15, 30 and 60 after baseline. 90- and 120-min measurements were included for studies in HFD-induced obese mice with impaired glucose tolerance. Total glucose AUC was calculated by trapezoidal rule using y=0 as baseline. Glucose readings were measured in mmol/L using the same ISO15197-compliant GlucoRx Nexus (GlucoRx, Guildford, UK) glucometer.

**Intraperitoneal insulin tolerance test**

Mice were fasted at 08:00. For acute assessment in lean mice, mice were administered either peptide (via subcutaneous injection) alone or peptide + Actrapid® (Novo Nordisk regular human) insulin at 14:00. The former group enabled an adjustment to account for the effect on insulin production from the peptide alone*. For chronic assessment in HFD-induced obese mice, mice were administered peptide at 12:00 and Actrapid® insulin at 14:00. For all studies, Actrapid® insulin was administered via intraperitoneal injection, with the injection volume adjusted to the body weight of the mouse such that each mouse received insulin at 0.75 U/kg. Blood glucose measurements were taken via tail venesection at 0, 15, 30, 60, 90 and 120 mins after baseline. Total glucose AUC was calculated by trapezoidal rule using y=0 as baseline. Glucose readings were measured in mmol/L using the same GlucoRx glucometer.

*This adjustment involved the following calculation: the average incremental change in blood glucose from baseline following peptide injection alone was subtracted from the blood glucose following peptide and insulin combinatorial injection. For example, if GIP108 alone reduced blood glucose from 8 mM at t=0 to 6 mM at t=15 (incremental change of -2 mM), and GIP108 plus insulin reduced blood glucose from 8 mM at t=0 to 4 mM at t=15, the adjusted glucose value at t=15 to account for the insulinotropic effect of GIP108 would be 6 mM [4 – (- 2)].

**Insulin bleeds**

Mice were fasted at 08:00. At 12:00, peptides were administered via subcutaneous injection. At 14:00, mice received an intraperitoneal injection with 20% glucose, with the injection volume adjusted to the body weight of the mouse such that each mouse received glucose at 2 g/kg. A blood sample was taken via tail venesection 15 minutes following glucose injection.

**Plasma assays**

Insulin was quantified following acute GIP108/NN-GIPR-Ant administration to lean mice using the Mercodia Mouse Insulin ELISA kit (10-1247-01, Mercodia, Uppsala, Sweden). Osteocalcin and CTX levels were quantified following chronic GIP108/NN-GIPR-Ant administration to HFD-induced obese mice. Plasma osteocalcin was measured using the Mouse Osteocalcin Assay (EEL003, Thermo Fisher, Waltham, MA, USA). Plasma CTX was measured using the Mouse CTX Assay (EEL219, Thermo Fisher, Waltham, MA, USA). Assays were performed according to the manufacturer’s instructions and were read using the SpectraMax i3x Microplate Reader (Molecular Devices, Silicon Valley, CA, US).

**Liver triglyceride assay**

Triglycerides were isolated from 175-225 mg of the left lobe of the liver (snap frozen). Livers were homogenised with ethanol proportional to liver weight (30 µL per 1 mg tissue), prior to 72-hour mixing, centrifugation and isolation of supernatant. Triglycerides were measured from the supernatant using Triglycerides TG enzymatic colorimetric Trinder assay serum plasma 3-1000 mg/dL single liquid stable reagent system (17624H, Sentinel Diagnostics, Milan, Italy), with the Clin Chem Calibrator lyophilised multiparameter calibration serum for clinical chemistry assays (16550, Sentinel Diagnostics, Milan, Italy) used as standard. Assays were performed according to the manufacturer’s instructions and were read using the SpectraMax i3x Microplate Reader.

**Oil red O staining**

Leftover sections of the left liver lobe (following liver triglyceride assay) were cryosectioned (10 µm thickness) and stained with Oil Red O (Sigma, Welwyn Garden City, UK)^3^. Slides were imaged using an EVOS M7000 microscope with a 10x objective.

**Adipose tissue gene expression**

RNA was isolated from 100 mg of inguinal adipose tissue biopsies (snap frozen) using a standard organic extraction method. cDNA was synthesised using a High Capacity Reverse Transcription cDNA kit (4368814, Applied Biosystems^TM^, Thermo Fisher Scientific, UK). Expression of hormone sensitive lipase (*Hsl*) and fatty acid binding protein 4 (*Fabp4*) was quantified using TaqMan^TM^ primer probes (Mm00495359_m1 and Mm00445878_m1, respectively, Applied Biosystems^TM^, Thermo Fisher Scientific, UK), with RT-PCR performed using the TaqMan^TM^ Fast Advanced Master Mix (4444557, Applied Biosystems^TM^, Thermo Fisher Scientific, UK), run on a QuantStudio 6 Flex real-time PCR system (Applied Biosystems^TM^, Thermo Fisher Scientific, UK). Gene expression was normalised to the housekeeping gene tyrosine 3-monooxygenase/tryptophan 5-monooxygenase activation protein zeta (*Ywhaz*) (Mm03950126_s1, Applied Biosystems^TM^, Thermo Fisher Scientific, UK).

**Islet isolation, dispersion and cADDis imaging**

Mouse pancreatic islets were isolated, dispersed, transduced with cADDis BacMam particles^4^ (Montana Molecular, MT, USA) and imaged as previously described^1^.

**Statistics**

Analyses were conducted using Prism 10.0 (GraphPad software). Unless stated otherwise, statistical comparisons were made between intervention group and vehicle control. Statistical tests used were one- or two-way ANOVA, mixed effect analysis (if missing values) and ANCOVA, as indicated in the figure legends. Dunnett's post-hoc test was used to correct for multiple comparisons. All summarised data points are presented as mean ± SEM. For dose response experiments, three-parameter fits were plotted. Statistical significance was taken at P<0.05.

**Supplementary Note**

The aim of this study was to provide a comprehensive metabolic evaluation of a GIPR agonist and GIPR antagonist at doses that were comparably anorectic in a HFD-induced obese mouse model. We first showed that GIP108 dosed at 100 nmol/kg, but not 10 or 30 nmol/kg, reduced food intake and body weight in a HFD-induced obese mouse model. Informed by doses selected by Yang et al^2^, we then compared the metabolic effect of daily administration of NN-GIPR-Ant (1.5 µmol/kg) versus GIP108 (100 nmol/kg) to HFD-induced obese mice. GIP108, but not NN-GIPR-Ant, reduced food intake and body weight. For the final 6 days of the study, we doubled the dose of NN-GIPR-Ant to 3 µmol/kg, and then observed significant food intake and body weight reduction. Finally, to confirm that NN-GIPR-Ant (3 µmol/kg) was able to reduce food intake acutely, we conducted a food intake study comparing the effects of single administration of GIP108 (100 nmol/kg) versus NN-GIPR-Ant (3 µmol/kg). Both peptides comparably lowered food intake compared to vehicle control. Thus, these were the doses selected for acute metabolic assessment in lean mice and chronic metabolic assessment in HFD-induced obese mice.

**References**

1. Davies I, Adriaenssens AE, Scott WR, et al. Chronic GIPR agonism results in pancreatic islet GIPR functional desensitisation. *Mol Metab*. 2025;92. doi:10.1016/j.molmet.2025.102094

2. Yang B, Gelfanov VM, El K, et al. Discovery of a potent GIPR peptide antagonist that is effective in rodent and human systems. *Mol Metab*. 2022;66. doi:10.1016/j.molmet.2022.101638

3. Mehlem A, Hagberg CE, Muhl L, Eriksson U, Falkevall A. Imaging of neutral lipids by oil red O for analyzing the metabolic status in health and disease. *Nat Protoc*. 2013;8(6):1149-1154. doi:10.1038/nprot.2013.055

4. Tewson PH, Martinka S, Shaner NC, Hughes TE, Quinn AM. New DAG and cAMP Sensors Optimized for Live-Cell Assays in Automated Laboratories. *J Biomol Screen*. 2016;21(3):298-305. doi:10.1177/1087057115618608

**Supplementary Figures**

**
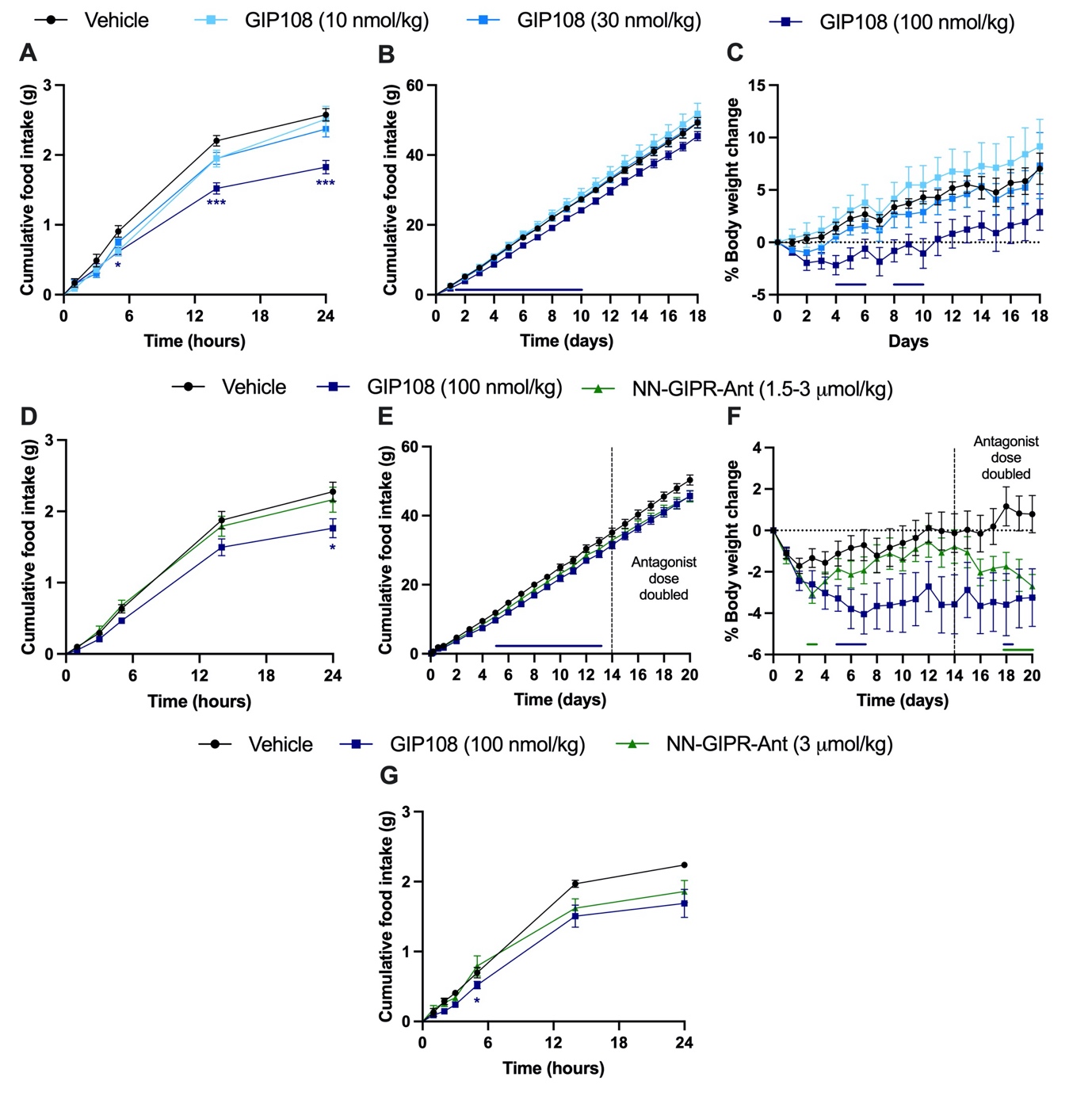
Supplementary Figure 1: Identifying anorectic doses of GIP108 and NN-GIPR-Ant in HFD-induced obese mice**

**A-C**) HFD-induced obese mice received a daily subcutaneous injection of saline, GIP108 (10 nmol/kg), GIP108 (30 nmol/kg) or GIP108 (100 nmol/kg) (n=5-8) for 18 days. **A**) Acute food intake (g) over 24 hours after first injection, **B**) cumulative food intake (g) over 18 days, **C**) % change in body weight over 18 days. **D-F**) HFD-induced obese mice received a daily subcutaneous injection of saline, GIP108 (100 nmol/kg) or NN-GIPR-Ant (1.5-3 µmol/kg) (n=7-8) for 20 days. For the first 14 days of the study, NN-GIPR-Ant was administered at 1.5 µmol/kg. For the remaining 6 days of the study, NN-GIPR-Ant was administered at 3 µmol/kg. **D**) Acute food intake (g) over 24 hours after first injection, **E**) cumulative food intake (g) over 20 days, **F**) % change in body weight over 20 days. **G**) Acute food intake (g) over 24 hours following injection of saline, GIP108 (100 nmol/kg) or NN-GIPR-Ant (3 µmol/kg) to HFD-induced obese mice (n=4-5). Food intake and body weight measurements over time have been analysed using a two-way ANOVA (**A**, **B**, **D**) or mixed effects analysis (**C**, **E**, **F**, **G**) with time and subgroup as covariables, with Dunnett’s test applied to correct for multiple comparisons against vehicle control. All values are displayed as mean ± SEM. Lines and stars indicate statistically significant differences. ∗ = P < 0.05, ∗∗ = P < 0.01, *** = P < 0.001, ∗∗∗∗ = P < 0.0001. The colour of the star or line denotes which group is statistically different compared to vehicle control.

**
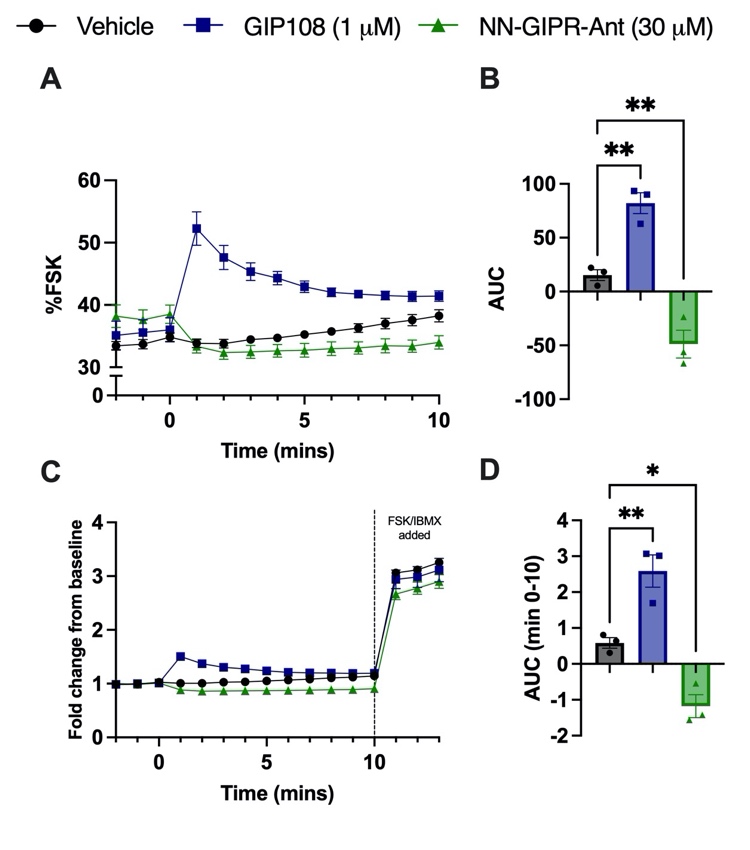
**

**Supplementary Figure 2: NN-GIPR-Ant reduces constitutive GIPR activity in dispersed mouse pancreatic islets**

**A, C)** cAMP responses in dispersed islet cells (transduced with cADDis) (n=3) following 10 minutes of peptide treatment and a further 3 minutes of forskolin (FSK)/ 3-isobutyl-1-methylxanthine (IMBX) treatment to induce a maximal response. In **A**) cAMP responses have been normalised to baseline and displayed as a percentage of the maximal FSK/IBMX response. In **C**) cAMP responses have been only normalised to baseline. **B, D**) AUC derived from corresponding cAMP time courses, analysed using a one-way ANOVA, with Dunnett’s test applied to correct for multiple comparisons against vehicle control. All values are displayed as mean ± SEM. ∗∗ = P < 0.01.


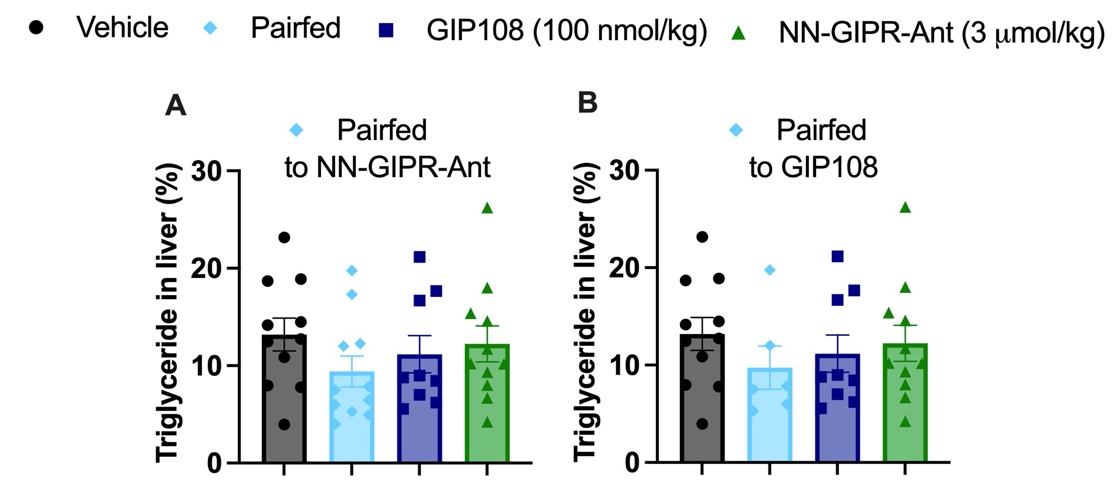


**Supplementary Figure 3: % Triglyceride in liver following chronic GIP108 or NN-GIPR-Ant treatment in HFD-induced obese mice.**

% Triglyceride in liver following daily afternoon subcutaneous injections of saline (n=11), GIP108 (100 nmol/kg) (n=9) and NN-GIPR-Ant (3 µmol/kg) (n=11) for 17 days. In **A**), 11 mice are pairfed to NN-GIPR-Ant treatment. In **B**), 6 mice are pairfed to GIP108 treatment.

**
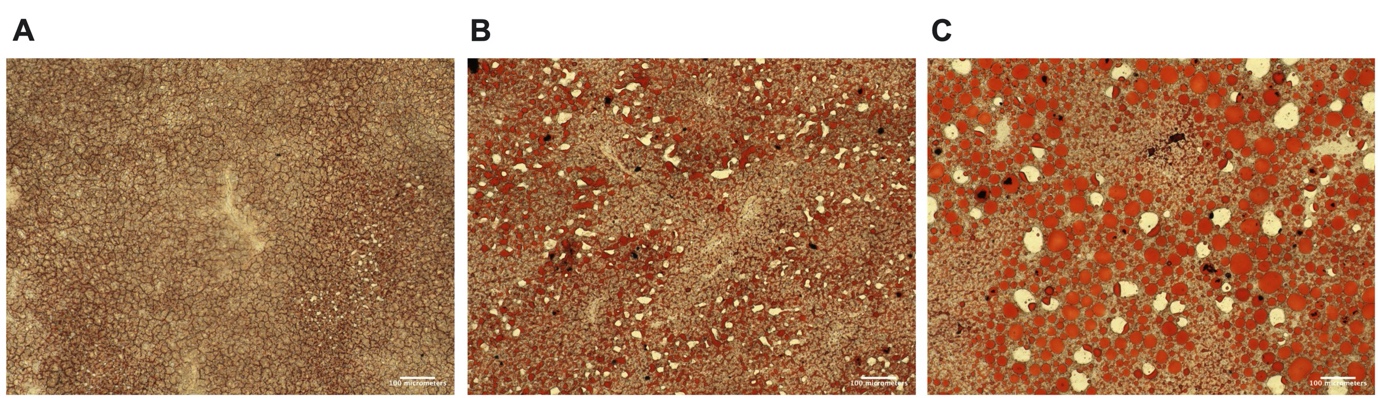
Supplementary Figure 4: Oil red O staining intensity reflects % liver triglyceride content**

Oil Red O staining of a liver with 3% triglyceride content (**A**), 10% triglyceride content (**B**) and 18% triglyceride content (**C**). Scale bar denotes 100 micrometres.

**
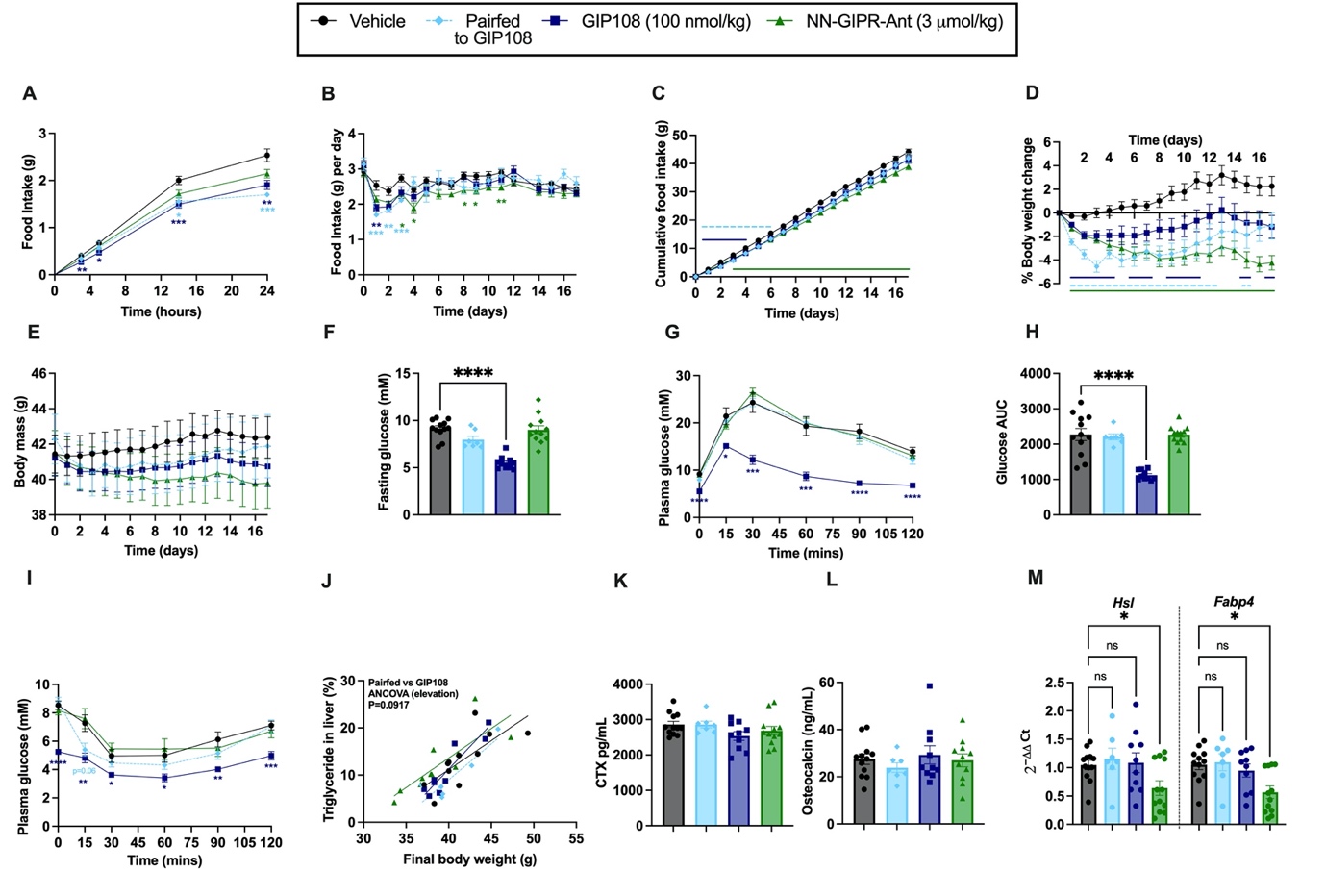
**

**Supplementary Figure 5: GIP108 and NN-GIPR-Ant both reduce body weight in HFD induced obese mice (Figure 3 continued)**

HFD-induced obese mice received daily afternoon subcutaneous injections of saline (n=12), GIP108 (100 nmol/kg) (n=10) and NN-GIPR-Ant (3 µmol/kg) (n=12) for 17 days. While an additional 12 mice were restrictively fed, 7 mice are included here such that they pair-feed to GIP108 whilst retaining a similar starting body weight. **A**) Acute food intake (g) over 24 hours after first injection, **B**) food intake (g) per day over 17 days, **C**) cumulative food intake (g) over 17 days, **D**) % change in body weight over 17 days, **E**) body mass (g) over 17 days, **F**) fasting glucose (mM) on day 13/14 2 hours after daily peptide injection, **G**) plasma glucose (mM) and **H**) glucose AUC measured during intraperitoneal glucose tolerance test on day 13/14, 2 hours after daily peptide injection with glucose administered at t=0, **I**) plasma glucose measured during an intraperitoneal insulin tolerance test on day 15/16, 2 hours after daily peptide injection with insulin administered at t=0, **J**) relationship between final body weight and triglyceride in liver (%) following 17 days of daily injection (1 outlier excluded per group), **K**) plasma CTX (pg/mL) and **L**) plasma osteocalcin (ng/mL) (1 outlier excluded for NN-GIPR-Ant), both measured following 17 days of daily injection, **M**) inguinal adipose tissue mRNA expression of hormone sensitive lipase (*Hsl*) and fatty acid binding protein 4 (*Fabp4*), expressed relative to housekeeping gene, measured following 17 days of daily injection. Food intake, body weight and plasma glucose measurements over time have been analysed using a two-way ANOVA with time and subgroup as covariables, with Dunnett’s test applied to correct for multiple comparisons against vehicle control. Gene expression has been analysed using a two-way ANOVA with gene and subgroup as covariables, with Dunnett’s test applied to correct for multiple comparisons against vehicle control. Fasting glucose, glucose AUC and plasma CTX/osteocalcin levels have been analysed using a one-way ANOVA with Dunnett’s test applied to correct for multiple comparisons against vehicle control. % triglyceride in liver has been analysed using ANCOVA between the pairfed group and GIP108 group, with final body weight as a covariable. All values are displayed as mean ± SEM. Lines and stars indicate statistically significant differences. ∗ = P < 0.05, ∗∗ = P < 0.01, *** = P < 0.001, ∗∗∗∗ = P < 0.0001. The colour of the star or line denotes which group is statistically different compared to vehicle control.
